# Differences in Soil Fungal Communities between European Beech (*Fagus sylvatica* L.) Dominated Forests Are Related to Soil and Understory Vegetation

**DOI:** 10.1371/journal.pone.0047500

**Published:** 2012-10-18

**Authors:** Tesfaye Wubet, Sabina Christ, Ingo Schöning, Steffen Boch, Melanie Gawlich, Beatrix Schnabel, Markus Fischer, François Buscot

**Affiliations:** 1 UFZ - Helmholtz Centre for Environmental Research, Department of Soil Ecology, Halle (Saale), Germany; 2 Fraunhofer Institute for Cell Therapy and Immunology, Department of Diagnostics, Leipzig, Germany; 3 University of Leipzig, Institute of Biology, Chair of Soil Ecology, Leipzig, Germany; 4 Max-Planck-Institute for Biogeochemistry, Department Biogeochemical Processes, Jena, Germany; 5 University of Bern, Institute of Plant Sciences and Botanical Garden, Bern, Switzerland; UC Irvine, United States of America

## Abstract

Fungi are important members of soil microbial communities with a crucial role in biogeochemical processes. Although soil fungi are known to be highly diverse, little is known about factors influencing variations in their diversity and community structure among forests dominated by the same tree species but spread over different regions and under different managements. We analyzed the soil fungal diversity and community composition of managed and unmanaged European beech dominated forests located in three German regions, the Schwäbische Alb in Southwestern, the Hainich-Dün in Central and the Schorfheide Chorin in the Northeastern Germany, using internal transcribed spacer (ITS) rDNA pyrotag sequencing. Multiple sequence quality filtering followed by sequence data normalization revealed 1655 fungal operational taxonomic units. Further analysis based on 722 abundant fungal OTUs revealed the phylum Basidiomycota to be dominant (54%) and its community to comprise 71.4% of ectomycorrhizal taxa. Fungal community structure differed significantly (*p*≤0.001) among the three regions and was characterized by non-random fungal OTUs co-occurrence. Soil parameters, herbaceous understory vegetation, and litter cover affected fungal community structure. However, within each study region we found no difference in fungal community structure between management types. Our results also showed region specific significant correlation patterns between the dominant ectomycorrhizal fungal genera. This suggests that soil fungal communities are region-specific but nevertheless composed of functionally diverse and complementary taxa.

## Introduction

Fungi are an important and highly diverse component of soil microbial communities. In forest ecosystems, they perform essential ecological functions including decomposition, element cycling, and are involved in biotic interactions such as mycorrhizal symbioses [1], [2]. Understanding factors shaping fungal diversity, community structure and spatial patterns is one of the central issues in soil microbial ecology [3]. The spatial distribution of soil fungal communities is thought to reflect their response to environmental factors such as soil nutrient availability [4], soil texture and water availability [5], and vegetation type [6], [7]. These factors are further controlled by geographic differentiation, changes in plant community composition due to forest management, and the associated impact on soil conditions [5], [8].

Studies on soil fungal diversity and community structure among geographic locations differing in soil, land-use or vegetation revealed contradictory results. For instance, Green et al [8] reported geographic differentiation in soil fungal community structure. In contrast, Kasel et al [5] only found weak regional differences, while land use within regions was important. Most studies in temperate forests focused on ectomycorrhizal fungi and root associated communities, where spatial variation in fungal communities was related to changes in soil environment, stand age, host tree species and herbaceous plant cover [7], [9], [10], [11], [12], [13]. Recently spatial heterogeneity of soil fungal communities in temperate forests was also reported to be explained by host tree species and soil environment [10]. Massively parallel high throughput pyrotag sequencing has recently been employed to assess soil fungal diversity [10], [14], [15], [16], [17]. Use of the same technique for large-scale soil fungal community analysis of geographically separated forest sites dominated by the same tree species could help to elucidate the relative contribution of geographic location, soil environment, forest management, and understory vegetation cover to soil fungal community structure.

In this context, we used a massively parallel high throughput pyrotag sequencing approach to analyze the soil fungal diversity and community structure of 9 managed and 9 unmanaged European beech (*Fagus sylvatica* L.) dominated forests distributed in three German regions. Previous culture based study on the distribution of yeasts [18] and 16S pyrotag based bacterial diversity [19] studies reported that soil microbial community composition differs between these study sites. Accordingly, we expected that the fungal community differs among the three study sites and between the management types within the study sites. Thus the main objectives of this study were to i) assess the influence of soil environment and understory vegetation related parameters on the fungal community structure, ii) evaluate the impact of forest management on fungal community composition within the study regions, and iii) assess correlations and patterns of co-existence among the dominant ectomycorrhizal fungal taxa.

## Materials and Methods

### Study Site and Sample Collection

This study was conducted as part of the Biodiversity Exploratories project ([20]; www.biodiversity-exploratories.de). The three exploratories, hereafter referred as study regions, are located in: (1) the Schwäbische Alb UNESCO Biosphere Area in Southwestern Germany; (2) the Hainich National Park and its surroundings (Hainich-Dün) in Central Germany; and (3) the Schorfheide Chorin UNESCO Biosphere Reserve in North-Eastern Germany. The study regions differ in climate, geology, and topographical situations and are representative for large parts of Central Europe (for details see Table 1 and [18]). We studied 18 20×20 m plots dominated by European beech (*Fagus sylvatica* L.). Within regions, the six studied plots were typically separated by several km separated from each other, and at least several hundred meters. In each of the three regions there were three age-class forests (characterized by homogeneous, even aged structure and intensive management), and three unmanaged forests (characterized by natural old-growth, uneven-aged stands with trees between 0 and 250 years old).

**Table 1:** Geographic location, main environmental characteristics, soil physical and geochemical parameters of the three regions.

			Management types	Plots	pH	Sand[g/kg]	Silt[g/kg]	Clay[g/kg]	C_org_[g/kg]	Nt[g/kg]	C:Nratio
Scchwäbische Alb	Location	SW Germany	Beech age-classforest managed	AEW04	6.4	70	534	396	78.5	6.0	13.1
		48° 34′ 58.8″ N, 9° 30′5.4′′ E		AEW05	4.5	47	587	368	57.5	4.5	12.9
	Elevation [m a.s.l.]	460–860		AEW06	5.4	107	575	318	39.1	3.2	12.4
	MAT [°C]	6–7	Beech unmanagedforest	AEW07	4.9	109	371	520	77.6	5.5	14.0
	MAP [mm]	700–1000		AEW08	5.1	34	296	670	105.0	6.8	15.5
				AEW09	6.4	56	495	449	60.0	4.5	13.4
											
Hainich-Dün	Location	Central Germany	Beech age-classforest managed	HEW04	6.2	28	665	307	79.4	5.8	13.7
		51° 1′ 17′′ N, 10° 30′36′′ E		HEW05	4.8	34	509	457	61.8	4.9	12.5
	Elevation [m a.s.l.]	285–550		HEW06	4.2	35	751	214	34.4	2.4	14.1
	MAT [°C]	6.5–8	Beech unmanagedforest	HEW10	4.8	34	481	485	67.6	5.1	13.3
	MAP [mm]	500–800		HEW11	4.8	30	566	404	58.5	4.5	13.1
				HEW12	3.9	46	790	164	31.1	2.0	15.3
											
Schorfheide Chorin	Location	NE Germany	Beech age-classforest managed	SEW04	3.2	874	101	25	33.2	1.6	20.5
		52° 57′ 0′′ N, 13° 37′0′′ E		SEW05	3.1	981	18	1	29.6	1.6	18.3
	Elevation [m a.s.l.]	3–140		SEW06	3.3	898	79	23	31.1	1.8	17.4
	MAT [°C]	8–8.5	Beech unmanagedforest	SEW07	3.2	881	119	1	24.3	1.5	16.3
	MAP [mm]	500–600		SEW08	3.1	743	237	20	29.2	1.8	16.1
				SEW09	3.0	937	45	18	23.0	1.4	16.6

MAT – Mean annual temperature, MAP – Mean annual precipitation.

**Figure 1:**
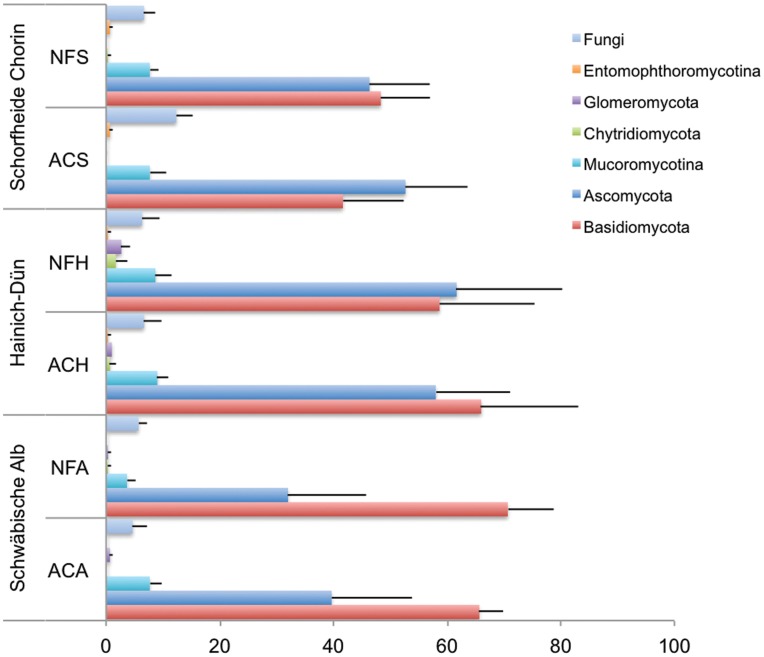
Relative distribution of the fungal phyla found in this study in the age-class (AC) and unmanaged natural (NF) forests in the Schorfheide-Chorin (S), Hainich-Dün (H), and Schwäbische Alb (A) study regions.

In April 2008 we took five soil samples per plot. First, the organic layer was removed with a quadratic 400 cm^2^ metal frame. Then, the mineral soil was sampled with a motor-driven soil column cylinder (diameter 8.3 cm, length 1 m) to obtain undisturbed cores (Fischer et al. 2010). After removing roots and stones, we pooled the A horizons of the five soil cores of each plot to obtain a representative composite sample and stored the samples at −20°C until molecular analysis. In addition, we determined organic carbon content (C_org_), total nitrogen content (Nt), pH and soil texture as described in [21] (see also Table 1).

**Figure 2:**
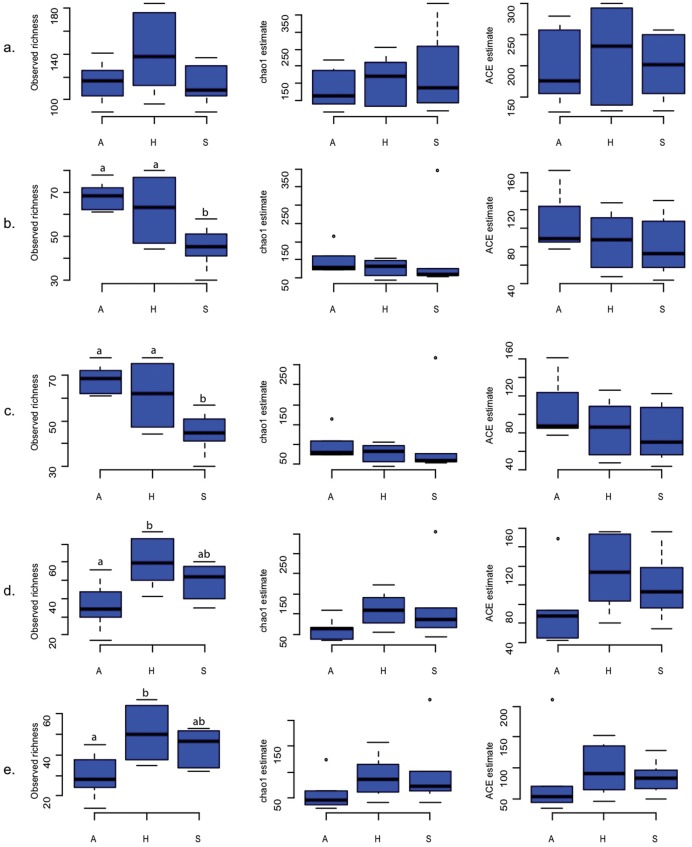
Observed and estimated (Chao1 and ACE) fungal richness across the three study regions, Schwäbische Alb (A), Hainich-Dün (H) and Schorfheide Chorin (S) presented using box plots for: (a) the fungal kingdom, (b) the phylum Basidiomycota, (c) the subphylum Agaricomycotina, (d) the phylum Ascomycota and (e) the subphylum Pezizomycotina. Different letters above bars indicate significant differences between the sites (*p*≤0.05) based on a Tukey *post hoc* pairwise comparison.

Furthermore, we recorded the vegetation of all plots in spring and in late summer of the same year. We identified all vascular plant taxa and estimated the percentage cover per species. Then we added up the percentage cover of all occurring species separately for two tree layers (5–10 m and >10 m), the shrub layer (0–5 m), and the herbaceous layer (including phanerophyte seedlings). In addition, we estimated the cover of bare soil, dead wood and litter on each plot. The spring vegetation data was used in this study. No significant variation of the soil and vegetation parameters was found among the management types in all the three study regions. The observed variation among the study regions is presented in the supplementary material (Fig. S1).

**Table 2:** Goodness of fit statistics or squared coefficients a of study sites, soil and understory vegetation parameters fitted to the Nonmetric Multi-dimensional Scaling (NMDS) ordination space of the fungal, Basidiomycotan, Ascomycotan, Agaricomycotan, Pezizomycotan, and Ectomycorrhizal (ECM) fungal communities.

	Fungi	Ascomycota	Pezizomycotina	Basidiomycota	Agaricomycotina	ECM fungi
Study regions^b^	0.867***	0.861***	0.839***	0.778***	0.774***	0.819***
Management types^c^	0.9156***	0.898***	0.875***	0.799***	0.791***	0.838***
pH	0.864***	0.887***	0.904***	0.715***	0.707***	0.786***
Sand	0.804***	0.882***	0.847***	0.640**	0.639**	0.747***
Silt	0.704***	0.685**	0.672***	0.556**	0.546**	0.657**
Clay	0.768***	0.780**	0.769***	0.658***	0.655***	0.728***
Corg	0.682**	0.716**	0.690***	0.628***	0.630**	0.682**
C:N ratio	0.804***	0.722***	0.696**	0.644**	0.647***	0.724***
Tree cover 1	0.119	0.081	0.086	0.042	0.071	0.042
Tree cover 2	0.057	0.101	0.088	0.040	0.045	0.003
Tree cover 1 and 2	0.029	0.044	0.026	0.011	0.026	0.052
Herb cover	0.613**	0.706***	0.688***	0.548**	0.559**	0.623***
Shrub cover	0.176	0.213	0.179	0.296	0.322*	0.342*
Herb and shrub cover	0.393*	0.511**	0.496**	0.444*	0.463*	0.506**
Bare soil	0.317	0.450*	0.354*	**0.317**	0.331**	0.489**
Dead wood cover	0.310	0.222	0.196	0.067	0.070	0.101
Litter cover	0.476**	0.576**	0.483**	0.204	0.212	0.380*

a significant squared root correlations are presented in bold (* p<0.05, ** p<0.01, *** p<0.001).

b the effect of study regions on the fungal community composition regardless of the management type.

c the effect of study region on the fungal community considering the management type.

Tree cover 1: Cover of 5–10 m high trees; Tree cover 2: Cover of >10 m high trees; Tree cover 1 and 2: cumulative tree cover of all trees.

**Figure 3:**
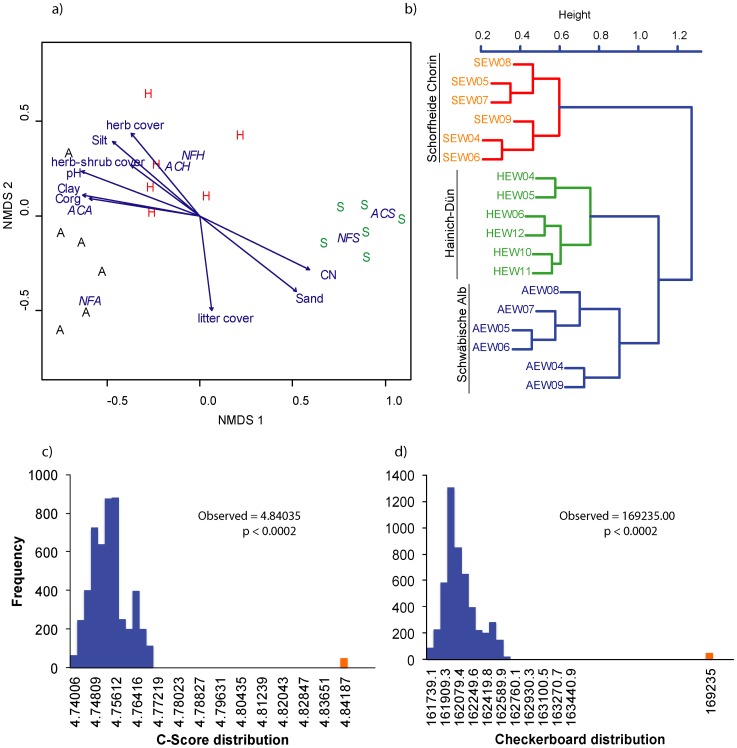
Fungal community structure across the three study regions: (a) NMDs ordination plot of the study regions Schwäbische Alb (A), Hainich-Dün (H), and Schorfheide Chorin (S), based on the fungal community composition identified as members of the fungal kingdom (Stress = 8.17). In each diagram soil and vegetation parameters used as an explanatory variable and found to be significant (*p*≤0.05) are represented as vectors. Management types are abbreviated as AC for age-class and NF for unmanaged natural forests followed by the respective study site. (b) Hierarchical cluster based on the most abundant fungal OTUs identified at least in four of the study plots where the fungal OTUs clearly separated the three regions. Fungal community assembly is nonrandom: C-score distribution (c) and checkerboard indices (d) for observed and randomized fungal OTU occurrence.

### DNA Extraction, Amplicon Library Preparation and Pyrosequencing

Soil microbial genomic DNA was extracted from 1 g of each composite sample using a MoBio PowerSoil DNA Isolation Kit (MoBio Laboratories Inc. Carlsbad, CA, USA), according to the manufacturer’s instructions. Fungal ITS rDNA amplicon libraries were produced using fusion primers designed with pyrosequencing primer B, a barcode and the fungal specific primer ITS1F [22] as a forward primer and pyrosequencing primer A and the universal eukaryotic primer ITS4 [23] as a reverse primer. We used a set of 10 bp MID-barcodes provided by Roche (Roche Applied Science). Amplicon libraries were produced from a pool of two dilution levels and three PCR replications. The PCR reactions were performed in a total volume of 50 µl reaction mix containing 1 µl DNA template, 25 µl Go Taq Green Master mix (Promega) and 1 µl 25 pmol of each of the two custom fusion primers. The reactions were performed using touchdown PCR conditions with an initial denaturation for 5 min at 95°C followed by: (1) 10 cycles of 94°C for 30 sec, 60–50°C for 45 sec (−1°C per cycle) and 72°C for 2 min; and (2) 30 cycles of 94°C for 30 sec, 50°C for 45 sec and 72°C for 2 min with a final extension step of 10 min.

**Table 3:** Relationships between the predictor variables pH, sand content and litter cover to the fungal community composition.

	pH			Sand			Litter cover		
	var	F	p	var	F	p	var	F	p
Fungi	**0.342**	**1.634**	**0.005**	**0.495**	**2.362**	**0.005**	**0.576**	**2.752**	**0.005**
Ascomycota	0.300	1.381	0.140	**0.430**	**1.975**	**0.005**	**0.563**	**2.586**	**0.005**
Pezizomycotina	**0.338**	**1.564**	**0.026**	**0.473**	**2.189**	**0.010**	**0.613**	**2.837**	**0.005**
Basidiomycota	0.300	1.381	0.065	**0.430**	**1.975**	**0.005**	**0.563**	**2.586**	**0.005**
Agaricomycotina	**0.408**	**1.750**	**0.015**	**0.588**	**2.523**	**0.005**	**0.656**	**1.816**	**0.005**
ECM fungi	**0.407**	**1.566**	**0.026**	**0.586**	**2.253**	**0.005**	**0.661**	**2.543**	**0.005**

Results show marginal tests using the dbRDA model, where var indicates the proportion of the fungal community variation explained by the predictor variable. Significant *p* values less than 0.05 are indicated in bold.

**Figure 4:**
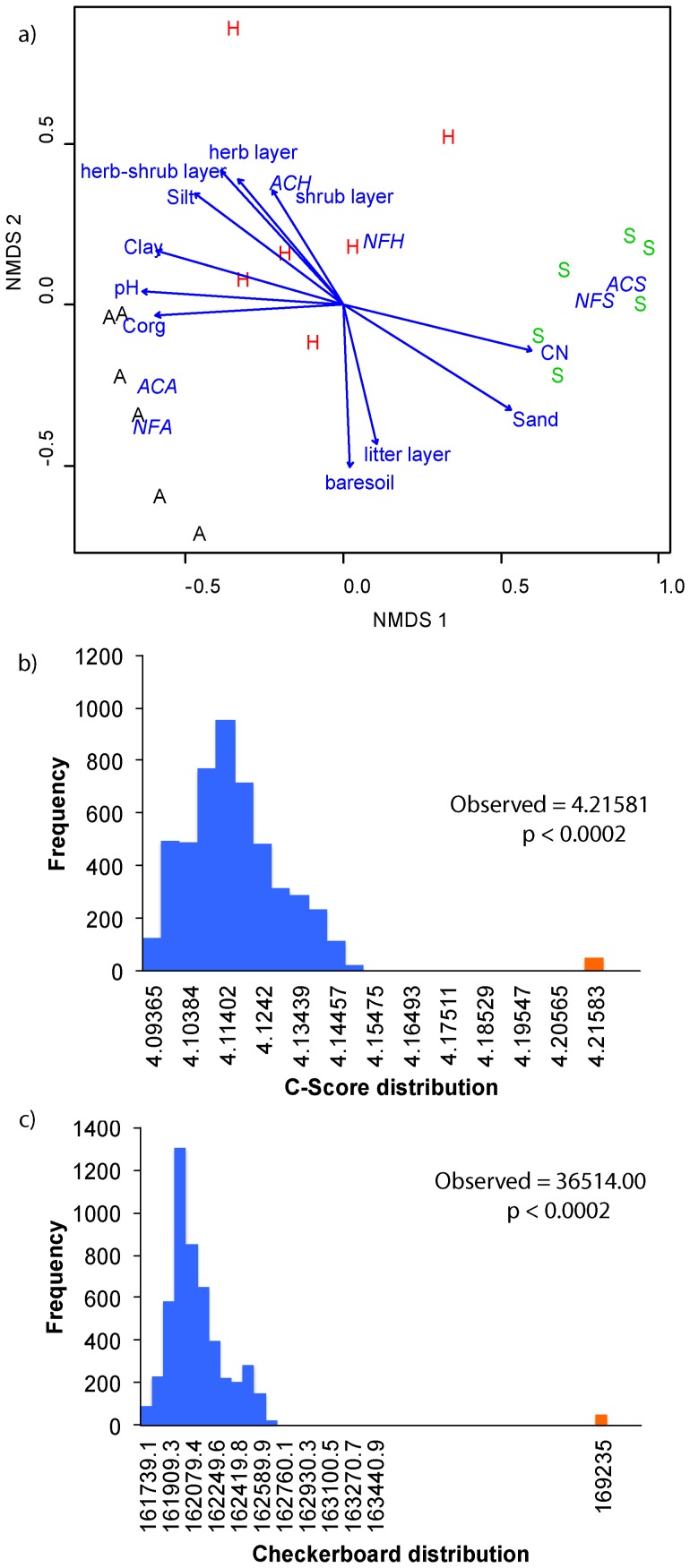
Ectomycorrhizal fungal community structure across the three study regions (a) NMDs ordination plot of the study regions Schwäbische Alb (A), Hainich-Dün (H), and Schorfheide Chorin (S), based on the ectomycorrhizal fungal community composition identified in this study (Stress = 11.34). Soil and vegetation parameters used as an explanatory variable and found to be significant (*p*≤0.05) are represented as vectors. Management types are abbreviated as AC for age-class and NF for unmanaged natural forests followed by the respective study site. ECM fungal community assembly is nonrandom: C-score distribution (b) and checkerboard indices (c) for observed and randomized ECM fungal OTU occurrence.

The PCR products were analyzed using a 1.5% agarose gel, equimolar volumes of amplified products from three positive amplicons of the six replicate PCRs per sample were pooled and gel purified using a Qiagen Gel Extraction Kit (Qiagen, Hilden, Germany). The amount of DNA in the purified amplicons was measured using a fluorescence spectrophotometer (Cary Eclipse, Agilent Technologies, Waldbronn, Germany). An equimolar mix of the 18 amplicon libraries was subjected to unidirectional pyrosequencing from the ITS1F end of the amplicons, using a 454 titanium amplicon sequencing kit and a Genome Sequencer FLX 454 System (454 Life Sciences/Roche Applied Biosystems, Mannheim, Germany) at the Department of Soil Ecology, Helmholtz Centre for Environmental Research (UFZ, Halle, Germany).

**Figure 5:**
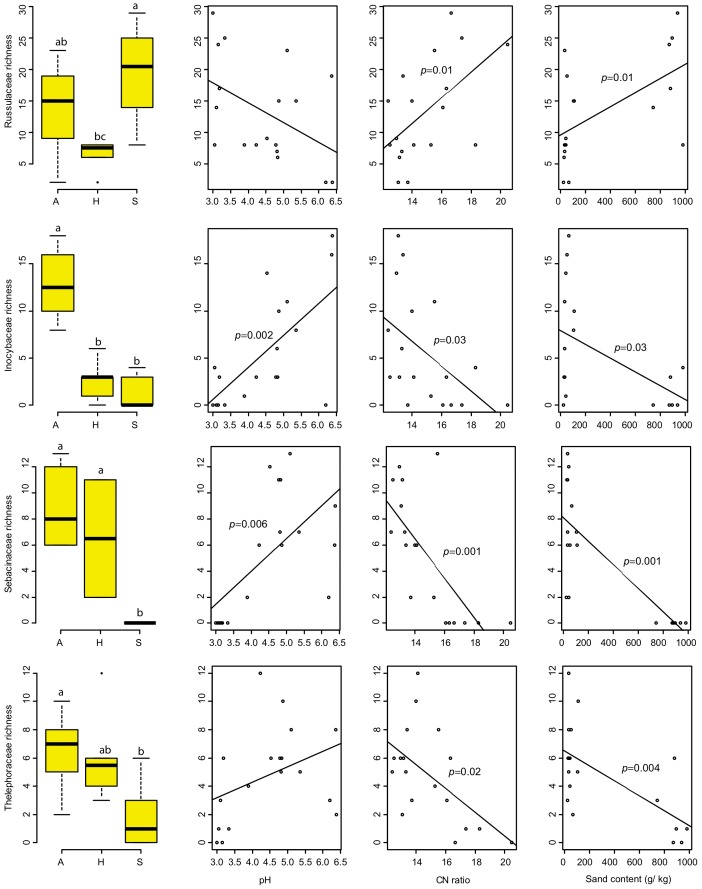
Relative distribution of the four dominant ectomycorrhizal fungal families *Russulaceae*, *Inocybaceae*, *Sebacinaceae* and Thelophoraceae among the three study regions and their relationships with soil pH, C:N ratio and Sand content determined using Box plots and linear regression analysis respectively. Different letters above bars in the box plots indicate significant differences between the sites (*p*≤0.05) based on a Tukey HSD *post hoc* pairwise comparison. Significant regression lines are presented with *p-*values.

### Bioinformatic Analysis

Multiple levels of sequence processing and quality filtering were performed. The 454 fungal ITS sequences were extracted based on 100% barcode similarity. Simultaneously sequence reads with an average quality score of <25, read length of <200 bp after trimming of the last 30 bps, ambiguous bases and homo-polymers of >8 bases were removed, barcodes and primers were trimmed using the split libraries script available in the Quantitative Insights In Microbial Ecology pipeline (QIIME) [24]. As our preliminary analysis showed that the sequence quality score drops below 20 after the 500^th^ bp of longer reads, sequences were trimmed to a maximum read length of 450 bp using mothur [25]. Sequences were then clustered and assigned to operational taxonomic units (OTUs) using the QIIME implementation of cdhit with a threshold of 97% pairwise identity after pre-filtering of sequences identical in the first 100 bases (n = 100). The representative sequences, the most abundant sequence in the respective clusters, were extracted and classified taxonomically according to the NCBI taxonomy based on a blast search against the NCBI nucleotide database excluding uncultured sequences and environmental samples followed by a 66% consensus level as implemented in CANGS [26]. A total of 1744 reads, which were not assigned to the fungal kingdom were cleared from the sequence dataset.

**Table 4:** Relationships among the ten dominant ectomycorrhizal (ECM) fungal genera determined using Spearman’s rank correlation a.

	Russula	Inocybe	Cortinarius	Phialophora	Sebacina	Lactarius	Elaphomyces	Xerocomus	Tomentella
Russula									
Inocybe	−0.054								
Cortinarius	−0.218	0.34							
Phialophora	−0.418	0.059	0.334						
Sebacina	−0.252	**0.783**	**0.492**	0.382					
Lactarius	0.093	0.396	0.223	−0.359	0.113				
Elaphomyces	0.303	−0.059	−0.421	−0.391	−0.323	0.444			
Xerocomus	0.069	**−0.47**	−0.156	0.186	−0.24	−0.339	−0.129		
Tomentella	0.095	0.395	0.324	0.309	**0.491**	0.011	−0.098	0.267	
Thelephora	0.005	0.098	0.403	0.108	−0.003	0.11	−0.196	0.29	**0.708**

a significant correlations with *p*<0.05 are in bold.

**Table 5:** Relationships between the dominant ectomycorrhizal fungal genera and soil and vegetation parameters determined using Spearman’s rank correlation a.

	Russula	Inocybe	Cortinarius	Phialophora	Sebacina	Lactarius	Elaphomyces	Xerocomus	Tomentella	Thelephora
pH	−0.267	**0.552**	0.431	**0.472**	**0.675**	−0.19	−0.348	−0.337	0.224	−0.088
Sand	0.452	−0.101	−**0.595**	**−0.783**	−0.442	0.304	0.382	−0.074	−0.302	−0.119
Silt	−0.441	0.275	**0.577**	**0.685**	**0.516**	−0.246	**−0.539**	0.232	**0.54**	0.399
Clay	−0.049	**0.483**	**0.544**	**0.492**	**0.741**	−0.137	−0.265	−0.279	0.398	−0.034
Corg	−0.259	0.404	0.467	**0.483**	**0.589**	−0.318	−0.315	−0.356	0.156	−0.173
C:N ratio	**0.527**	**−0.524**	−**0.682**	**−0.574**	**−0.79**	−0.045	0.426	0.277	−0.414	−0.21
Tree layer 1	−0.368	−0.125	−0.072	0.258	0.183	−0.46	−0.207	−0.229	0.095	−0.129
Tree layer 2	0.179	−0.013	−0.043	−0.365	−0.132	0.02	0.038	−0.099	0.019	0.036
Tree layers 1 and 2	−0.044	−0.089	−0.064	−0.022	0.054	−0.216	−0.103	−0.236	0.095	−0.058
Herb layer	−0.384	0.191	**0.578**	**0.842**	0.461	−0.21	−0.46	0.206	0.456	0.323
Shrub layer	−0.179	0.151	**0.599**	0.452	0.43	−0.191	**−0.555**	0.201	0.277	0.296
Herb and shrub cover	−0.269	0.265	**0.615**	**0.53**	0.458	−0.046	**−0.512**	0.255	0.397	0.436
Bare soil	0.314	**0.478**	−0.07	**−0.522**	0.119	0.385	0.445	−0.46	−0.062	−0.098
Dead wood cover	−0.173	−0.272	−0.07	0.259	−0.026	**−0.482**	−0.427	**0.552**	−0.1	−0.157
Litter cover	0.382	**0.542**	0.125	**−0.58**	0.296	0.355	0.298	−0.459	0.161	0.006

a significant correlations with *p*<0.05 are in Bold.

Tree cover 1: Cover of 5–10 m high trees; Tree cover 2: Cover of >10 m high trees; Tree cover 1 and 2: cumulative tree cover of all trees.

To facilitate screening of potentially chimeric sequences, we established a reference fungal ITS rDNA sequence database, consisting of fungal ITS rDNA sequences downloaded from the NCBI GenBank database, screened for sequences with a minimum length of 400 bp, pre-filtered at 98% identity and clustered at a 97% similarity using cdhit as implemented in QIIME. A total of 6601 sequences representing individual clusters were aligned using MAFFT [27] and used as core alignment sets. Representative sequences of OTUs of the fungal ITS pyrotags assigned under the fungal kingdom were aligned with the custom ITS reference dataset and checked for chimeras using the chimera uchime algorithm, as implemented in mothur. Subsequently a total of 6222 sequences including potentially chimeric reads and having less than 90% alignment length to the fungal reference database were removed from the sequence dataset.

Finally we found 29169 fungal sequences, which were grouped into 2271 OTUs. However, the number of reads per sample was variable ranging from 856 to 2505. Thus the number of sequences per sample was normalized to the smallest sample size by random removal of sequences using the normalized.shared command as implemented in mothur.

### Statistical Analyses

Data were analyzed using R, version 2.13.2 [28]. All the statistical analyses were carried out using the sequence count within each OTU as abundance value [29] of the non-singleton fungal communities. The observed richness and the Chao1 and ACE estimates of the fungal communities were calculated using the estimateR function of the vegan package [30]. Diversity of the fungal community was assessed calculating Shannon-Wiener, invSimpson, and rarified diversity indices. Differences in the fungal diversity were compared using ANOVA followed by Tukey *post hoc* test. Similarity in the fungal community structure among the three regions and between the management types within each study region was analyzed using the functions ANOSIM and adonis of the vegan package respectively.

Relationships between fungal communities with environmental variables, which include study site, management type, and soil and vegetation parameters, were visualized using non-metric multidimensional scaling (NMDS) on the basis of Bray-Curtis distance matrix using the nmds function of the labdsv package [31]. The envfit function of vegan was used to fit a centroid to each level of the environmental variables. The function envfit calculates the goodness of fit statistics or squared correlation coefficient value as a measure of separation among the different levels of the variables. Significance or empirical *p* value of each variable was calculated using 999 random permutations. Model of multivariate analysis of variance was constructed using distance-based redundancy analysis (dbRDA) based on the Bray–Curtis distance using the function capscale of vegan to determine the most influential environmental variables on the fungal community composition. Marginal tests were performed to test the amount of variation explained by the selected variables. Significance tests were performed through non-parametric permutation, which do not rely on the assumption of multivariate normality [32].

In order to assess the influence of the low abundant fungal OTUs on the fungal community structure we performed a hierarchical cluster analysis. Before clustering, fungal OTUs were sorted by the sum of their abundance across all samples and less abundant OTUs were excluded from the analysis by maintaining those OTUs occurring at least in four samples. Samples were then hierarchically clustered using the complete linkage clustering criteria of the hclust function of vegan using Spearman rank’s similarity metrics [33].

The fungal community assembly rule was tested using the C-score, the tendency for species to exclude one another from a given niche and checkerboard indices that corresponds to the number of species pairs that never co-occur [34] were determined using a null hypothesis of random community assembly. To assess the significance of the scores obtained from the datasets 5,000 matrices were randomly generated from the fungal and putative ectomycorrhizal fungal OTU datasets with EcoSim Version 7.0 [35]. C-score and checkerboard distributions and *p* values were determined from the simulations using EcoSim’s default settings. Furthermore, we used Spearman rank correlation tests to elucidate the relationship between the environmental parameters and the dominant ectomycorrhizal fungal communities at the Family and Genus level. The same analysis was also used to test the patterns of co-occurrence among the ECM fungal communities. Both spearman’s correlation coefficients and their significance values were calculated using the function corr.test of the psych package [36]. Based on preliminary normality tests, both species and environmental variables were log-transformed.

## Results

### Sequence Quality Control and Characterization

A total of 37135 reads were obtained after the first sequence processing and quality filtering steps. From this amount a total of 7966 reads were removed including 1744 non-fungal and 6222 potentially chimeric sequences. The remaining 29169 fungal sequences were distributed unevenly ranging from 856 to 2505 reads per sample. The number of sequences per sample was therefore normalized to 856 reads leading to 15408 fungal ITS sequences clustered into 1655 fungal OTUs. The 722 abundant OTUs excluding singletons were finally used for further analyses in order to address our questions.

Taxonomic assignment of the abundant fungal OTUs found in this study indicated members of the phylum Basidiomycota (390 OTUs/54%) as the most dominant, followed by Ascomycota (261 OTUs/36.1%), Mucoromycotina (24 OTUs/3.3%), Glomeromycota (7 OTUs/1%), Chytridiomycota (4 OTUs/0.6%) and Entomophthoromycotina (1 OTU/0.1%). The remaining 35 OTUs (4.8%) were assigned to the kingdom level according to the NCBI nucleotide taxonomy and represented unknown or un-annotated environmental sequences (Figure 1). In general the vast majority of the fungal OTUs were assigned to the major taxonomic levels of subphylum (647 OTUs, 89.6%), class (622 OTUs, 86.2%), order (624 OTUs, 86.4%), Family (567 OTUs, 78.5%) and genus (583 OTUs, 80.6%).

### Fungal Diversity

Comparison of diversity showed that the relative soil fungal distribution at the phylum level differed between the management types within a study region particularly in Schorfheide-Chorin and Hainich-Dün and also among the three regions (Fig. 1). For example, the soils of the Schwäbische Alb region were characterized by a higher percentage (59.8%) of Basidiomycota compared to those of the Hainich-Dün (49.3%) and Schorfheide-Chorin (42.9%). On average, the soils in forests of Schorfheide-Chorin were characterized by a higher percentage of Ascomycota (43.6%) compared to those of the Hainich-Dün (39.1%).

ANOVA followed by a Tukey *post hoc* pair wise comparison showed no significant difference in the observed and estimated OTU richness among the three regions. In contrast, similar tests showed that the observed Basidiomycotan, Agaricomycotan, Ascomycotan and Pezizomycotan richnesses differed significantly (*p*≤0.05) among the three regions. The Schwäbische Alb and Hainich-Dün regions exhibited significantly higher basidiomycotan and agaricomycotan OTU richnesses than Schorfheide-Chorin (Figure S2).

In all three taxonomic levels tested, extrapolative species richness estimates (chao1 and ACE estimators) were more than double compared to the observed richness, indicating the presence of highly diverse fungal communities in these forest ecosystems (Figure 2). The Shannon-Wiener diversity index ranged from 3.0 to 4.7, also indicating a diverse fungal community, especially in the Hainich-Dün forest soils. The invSimpson and rarified species diversity indices also followed a similar pattern (Figure S2).

### Fungal Communities and Factors Explaining the Community Structure

Analysis of the similarity of fungal communities using ANOSIM revealed a significant (R = 0.783, *p*≤0.001) variation among the three regions. Consistently, fitting of the study site to the NMDS ordination plot indicated significant differences in the fungal community structure among the study regions at the kingdom, phylum and subphylum levels (r^2^≥0.75, *p*≤0.001, Table 2). The soil physical and chemical parameters were also significantly related to differences in the fungal community composition. Among the vegetation parameters, the cover of the herbaceous layer influenced significantly the fungal community structure in all three taxonomic levels tested, where increased cover of the herbaceous layer was related to an increase in the fungal communities (Figure 3a, 4a and Figure S5).

The dbRDA model analysis indicated soil pH, sand content, and litter cover to be the most important factors shaping the fungal community composition (F = 3.925, *p* = 0.005, Table 3). Marginal tests, however, showed that sand content and litter cover were significantly related to the fungal community composition (pH - F = 1.381, *p* = 0.065, Sand - F = 1.975, *p* = 0.005, litter cover - F = 2.586, *p* = 0.005). The litter cover was obviously a driver of the fungal community structure, which was mainly reflected by its positive correlations to the Ascomycotan (*p*≤0.01, Table 2, Figure S5) and ectomycorrhizal fungal communities (*p*≤0.01, Table 2, Figure 4a).

The observed change in fungal community composition among regions was also found when we compared datasets for the two management types separately (see Table 2). Overall, the number of shared fungal OTUs between age-class and unmanaged forests within the study regions ranged between 37% and 50% (Figure S3). However, the contribution of management to variation in fungal community composition within the study regions was not significant (adonis, R^2^ = 0.049, *p* = 0.61). Hierarchical cluster analysis based on the most abundant OTUs occurring at least in four of the study samples also separated the fungal communities into three major groups corresponding to the study regions (Figure 3b), which consistently demonstrated that the fungal community structure differs among the three regions but not between the management types within the study regions.

The fungal community co-occurrence test found that the C*-*score for the real fungal OTU dataset was 4.84, which is significantly greater than the simulated mean C*-*score of 4.75 obtained from the simulated dataset (*p*<0.0001). The checkerboard measure for the fungal communities (169235.00) was also significantly greater than the simulated mean checkerboard measure (162069.70, *p*<0.0001, Figures 3c and 3d). These results indicate that the fungal community in these forest ecosystems is composed of non-randomly assembled but interacting fungal communities.

### Correlation between ECM Fungal Communities and Environmental Parameters

We were able to categorize 328 (47.2%) of the abundant fungal OTUs as mycorrhizal fungi. This includes 7 arbuscular mycorrhizal (AM) and 320 putative ectomycorrhizal (ECM) fungal taxa based on literature [37], [38], [39]. Members of the phylum Basidiomycota (83.5%) were the dominant ECM fungal communities, representing 16 families and 27 genera. The remaining 10 families and 17 genera were members of the Phylum Ascomycota. The most abundant ECM fungal families were *Russulaceae*, *Inocybaceae*, *Boletaceae*, *Sebacinaceae*, *Cortinariaceae*, Calvulinaceae, *Thelophoraceae*, *Magnaporthaceae, Pyrenomataceae*, and *Entholomataceae*. The genera *Russula*, *Inocybe, Cortinarius, Phialophora, Sebacina, Lactarius, Elaphomyces, Xerocomus, Tomentella*, *and Thelephora* were the ten dominant ectomycorrhizal fungal genera (Supporting information Table S1).

Analysis of ECM fungal community structure using ANOSIM revealed significant (R = 0.798, *p*≤0.001) variations among the three regions. NMDS ordination analysis followed by fitting of the environmental variables also confirmed that the ECM fungal community composition differed significantly among the three regions (r^2^ = 0.819, *p*≤0.001) and was influenced by soil physico-chemical parameters (Figure 4a, Table 2). The dbRDA model analysis for the ECM fungal communities found that soil pH, sand content and litter cover to be the most important factors (F = 3.598, *p* = 0.005, Table 3), where all the three variables were significantly related to the community composition (pH - F = 1.566, *p* = 0.026, Sand - F = 2.253, *p* = 0.005, litter cover - F = 2.543, *p* = 0.005).

ANOVA followed by a Tukey HSD *post hoc* analysis indicated significant variation in ECM fungal family richness among the three regions. Similar analysis of individual families also revealed a significant variation in the richness of some families among the study regions and a significant correlation to soil parameters. For example richness of the *Russulaceae* was significantly lower in the Hainich-Dün site (p≤0.05) compared to the highest richness in Schorfheide Chorin, where the observed richness was positively correlated with sand content and soil C:N ratio. The *Sebacinaceae* and *Thelephoraceae* showed relatively low richness in Schorfheide Chorin. Their richness was increasing with soil pH and decreasing with sand content and soil C:N ratio. However, richness of members of the *Inocybaceae* was increasing with soil pH. Their diversity was higher in the Schwäbische Alb than in both other regions (Figure 5).

Consistent with the general fungal community structure, the observed change in ECM fungal community composition among regions was also found when we compared datasets for the two management types separately (see Table 2). But, we found no statistically significant effect of management within the respective study sites (adonis, R^2^ = 0.043, p = 0.779). However, the relative distribution of the ECM fungal communities showed different patterns between the two management types particularly within the Schwäbische Alb and Schorfheide Chorin regions (Figure S4).

The C-Score and checkerboard pairs analysis of ECM fungal co-occurrence also showed that both the observed C-Score of 4.22 and checkerboard measure of 36514.00 were significantly higher than the randomized datasets (C-Score = 4.11, *p*<0.0001 and checkerboard = 34850.89, *p*<0.0001, Figures 4b and 4c) that the ectomycorrhizal fungal community is composed of non-random fungal communities. Spearman’s rank correlation analysis of the 10 most abundant ECM fungal genera among the study plots revealed some significant positive and negative correlations (Table 4). For example the genus *Tomentella* showed a strong positive correlation with *Thelephora* and *Sebacina*. The genera *Inocybe* and *Sebacina* on the other hand showed strong correlations with other ECM genera. *Inocybe* was significantly and positively correlated with *Sebacina* and negatively with the genus *Xerocomus,* whereas *Sebacina was* significantly and positively correlated with *Cortinarius*. Similar analysis within the individual study regions (Table 5) also showed region-specific correlation patterns consistent with the observed correlations of the fungal genera with the soil and vegetation parameters. Our results in general demonstrate that the observed differences in ECM fungal community structure among and within the three regions were rather characterized by the co-occurring and potential functional complementarity of ECM fungal taxa.

## Discussion

### Sequence Analysis

Pyrosequencing in microbial ecology has strongly improved resolution of community studies by increasing the numbers of sequences per sample compared to traditional cloning and sequencing approaches. A number of microbial diversity studies filtered out sequences by removing noisy and chimeric reads, thus reducing the bias and enabling documentation of large numbers of singletons as members of the “rare biosphere” [40], [41], [42], [43], [44]. However, most of the analyses in recent fungal diversity studies have focused on abundant sequence types (OTUs), while the ecological significance of singletons as rare biosphere is still being debated. Some authors recommend excluding all singletons from the analysis [16], [17], while others consider them as an evidence for a large unexplored diversity [15], [45].

In this study we were able to screen a total of 1655 fungal OTUs including 933 singletons. The vast majority of the singletons (62%) showed ≥97% identity at a minimum of 90% query coverage to sequences available in the NCBI GenBank database; the remaining 38% may correspond to the unexplored and rare fungal biosphere. We also observed that 287 (30.8%) of the singletons were non-singleton OTUs before the normalization step and 82% were taxonomically assigned to the Genus level. These observations provide evidence against the current practice to exclude singletons in general with the assumption that they are non-biological in nature [17]. However, we presented results based on the abundant fungal OTUs excluding singletons, although all the statistical analysis performed including the singletons showed similar results.

### Fungal Diversity and Community Structure Among Study Regions

In this study we found a huge soil fungal diversity in the soils of the investigated European beech forests with clear differences in community composition among the three study regions located in North, Central and South Germany. The fungal community was dominantly composed of Basidiomycota followed by Ascomycota, which is in accordance with previous studies investigating temperate forest soils [10]. Noteworthy, significant differences in the observed fungal richness between the study regions were found at the phylum (Basidiomycota, Ascomycota) and subphyla (Agaricomycotina and Pezizomycotina) levels (Figure 2, Figure S2).

In general the fungal community structure was found to be significantly different among the three study regions. The NMDS ordination plot and ANOSIM using the abundant fungal OTUs and the hierarchical clustering using only the most abundant fungal OTUs occurring at least in four samples showed consistent clustering of the three study regions, indicating a consistent pattern of fungal community structure. Having in mind that the three study regions are distantly located in the North - South gradient across Germany, our finding is in line with the report of Green et al [8] who studied Ascomycotan fungal communities in four distinct land use systems in arid Australia that varied substantially in geology, topography and native vegetation. They analyzed a total of 1,536 soil samples with distances ranging from 1 m to 100 km using a pair wise sample comparison and found a strong differences in the structure of soil fungal communities among geographic distances, which contradicts the weak region effect on fungal community structure found by Kasel et al [5] who studied soil fungal communities among four land uses located across three regions separated by distances of 50, 175 and 215 km in central Victoria, south-eastern Australia.

### Relationships of Fungal Community Structure with Soil Physico-chemical Parameters

Differences in fungal community structure may result from different abiotic factors. A number of studies reported effects of soil physical and chemical parameters on fungal community structures [7], [15], [46]. We also found that the fungal communities were positively correlated to the first NMDS axis with the soil sand content and C:N ratio, while negative correlations were found with pH, C_org_, silt, and clay content (Figure 3a). The dbRDA based model indicated sand content and pH as the two important soil parameters explaining the fungal community composition (Table 3). Compared to both other regions soil pH, C_org_, clay, and silt contents were significantly lower in Schorfheide-Chorin, while the C:N ratio and sand content were significantly higher at this site (Figure S1). The patterns of the NMDs ordination plot and the strong correlation with the soil parameters were consistent among tested taxa, whereby the Hainich-Dün and Schwäbische Alb regions were relatively close to each other indicating similar soil conditions.

### Relationships of Fungal Community Structure with Vegetation and Litter Cover

We found evidence that vegetation characteristics, mainly the herbaceous plant and litter covers, affect the diversity and community composition of soil fungal communities in these European beech dominated forests. Our data indicates that the herbaceous plant cover was significantly correlated with the soil fungal communities, which corroborates the report of Burke et al [7], who analyzed ECM roots from soil cores in a Mature Beech-Maple forest and found a strong correlation between root fungal communities and the herbaceous plant cover. They also found a strong correlation of the herbaceous layer with the genus *Russula*. Our data, however, showed that presence and relative abundance of the genera Cortinarius and, *Phialophora* correlated positively with the herbaceous plant cover. On the other hand the genera *Phialophora* and *Inocybe* showed a strong negative and positive correlation with the litter cover, respectively. This partially reflects the observed strong and negative correlation between herbaceous plant and litter cover in these beech forest systems (r = −0.642, *p* = 0.004) and their impact in the fungal community. However, the functional links underlying these correlations remain to be investigated.

### Management Effects on Fungal Community Structure

It has been well documented that land use changes influence the composition of soil fungal communities indirectly by changing plant and soil properties, but such evidence presented previously has focused on comparisons between different dominant tree species [6], or near-natural vs. plantation forests [5]. In our study we found no influence of management on the fungal community structure within the study sites. Remarkably, we also found no effect of management on the soil parameters and vegetation cover within the study regions, which could partly explain the absence of management effect on the soil fungal community structure. However, although it is not statistically significant, the relative distribution of the fungal communities as exemplified by the ECM fungal families indicated differences between managed and unmanaged forest stands in the respective study regions (Figure S3). The high degree of specificity of fungal communities, especially ECM fungi to their host plants under similar edaphic conditions [11], [47], could also lead to the observed weak impact of management on the fungal community structure.

### Correlation and Co-existence of Ectomycorrhizal Fungi

Consistent with the total fungal community structure, the ectomycorrhizal fungal communities also differed significantly between the three regions, but no management effect was found within the study sites. The ECM fungal communities were dominated by the ECM genera *Russula*, *Inocybe*, *Cortinarius*, *Lactarius, Thelephora,* and *Tomentella*, which were also reported to be the most diverse and abundant groups of ECM fungi in a number of studies [13], [39], [48], [49]. Besides the significant variation on the ECM fungal community structure among the study regions in response to the soil physico-chemical parameters (Figure 4a and Figure 5), we observed strong study region specific correlations between the ECM fungal genera, suggesting different patterns of co-existence. This is in line with previous studies that reported co-existence of ECM fungi in colonized roots [7], [13]. The observed non-random patterns of co-occurrence, differences in the identity and number of correlated ECM fungal genera reflect the degree of variation in environmental preferences and niche partitioning [11], [12].

### Conclusions

The use of pyrosequencing in microbial ecology is a powerful tool and increasingly becomes a standard method, where strict quality filtering [44] and sequence normalization [5050] are crucial analysis steps. Although we presented our data based on abundant fungal OTUs, we found comparable results including singletons in the analysis. The observation that about 30% of the singletons were abundant OTUs before the sequence read normalization step suggests the need for methods to screen singletons in order to assess the contribution of the rare (or rarely detected) fungal biosphere in a given ecosystem, especially when considering temporal variations.

In general, in this study, based on ANOSIM of the abundant fungal OTUs and hierarchical cluster analysis of the most abundant fungal OTUs occurring at least in four samples, we found consistent results showing that the fungal community structure differs among the study regions. Our results also indicate that soil fungal community composition is mainly influenced by soil physico-chemical parameters and the herbaceous plant and litter cover, where soil pH, sand content and litter cover explained most of the variation in the fungal community composition. However, the forest management showed a very weak impact in the studied forests dominated by the same tree species. This suggests the need for large-scale biogeographic studies with ecologically broader sampling and analysis of soil fungal communities in order to find the functional relationship of biotic and abiotic parameters varying with forest management. Our study also demonstrated ECM fungal correlation patterns that differ between the three beech forest sites, supporting the functional diversity and complementarity of ECM fungi. Future research on comparative study of root and soil ECM fungal communities coupled with isolation and functional analysis of the dominant taxa is needed to explore functional links.

## Supporting Information

Figure S1 Soil and understory vegetation parameters among the three study regions depicted using box plots. Schwäbische Alb (A), Hainich-Dün (H) and Schorfheide Chorin (S) study sites. Different letters above bars indicate significant differences between the sites (*p*≤0.05) based on a Tukey *post hoc* pairwise comparison.
(TIF)

Figure S2 Rarefied species richness, Shannon and invsimp diversity indices across the three study regions, Schwäbische Alb (A), Hainich-Dün (H) and Schorfheide Chorin (S), for: (a) the fungal kingdom, (b) the phylum Basidiomycota, (c) the subphylum Agaricomycotina, (d) the phylum Ascomycota and (e) the subphylum Pezizomycotina. Based on a Tukey *post hoc* pairwise comparison at *p*≤0.05 no significant differences were found.
(TIF)

Figure S3 Distribution of shared and unique fungal OTUs among the two management types of the three study regions (a) age class beech forests (b) among the unmanaged beech forests, and between the two management types of the study regions Schwäbische Alb (c), Hainich-Dün (d) and Schorfheide Corin (e).
(TIF)

Figure S4 Relative distribution of ectomycorrhizal fungal families between the two management types in the three study regions.
(TIF)

Figure S5 NMDs ordination of the study sites Schwäbische Alb (A), Hainich-Dün (H) and Schorfheide Chorin (S), based on the fungal community composition identified as members of the phylum Ascomycota (Ascomycotan communities, stress = 10.66), phylum Basidiomycota (Basidiomycotan communities, stress = 12.66), subphylum Pezizomycotina (Pezizomycotan communities, stress = 10.73) and subphylum Agaricomycotina (Agaricomycotan communities, stress = 12.80). Soil and plant parameters used as an explanatory variable and found to be significant (*p*≤0.05) are represented as vectors. The two management types are presented as AC  =  age class and NF  =  unmanaged beech forests followed by the respective study site abbreviations.
(TIF)

Table S1 Ectomycorrhizal fungal community distribution. Putative ECM fungal families and genera found in this study and the study sites and management types they are found. (Numbers refer to the number of fungal OTUs of the respective fungal family found in the respective management type). Abbreviations: AC  =  age class and NF  =  unmanaged beech forest. Note: For Ascomycetes fungal taxa without a clearly defined family classification we used the genus names in the family column.
(DOCX)
