# Supplementary figures and images for: Differences in Soil Fungal Communities between European Beech (Fagus sylvatica L.) Dominated Forests Are Related to Soil and Understory Vegetation
Source: PLoS One. 2012 Oct 18;7(10):e47500. doi: 10.1371/journal.pone.0047500 (PMC3475711; doi:10.1371/journal.pone.0047500)

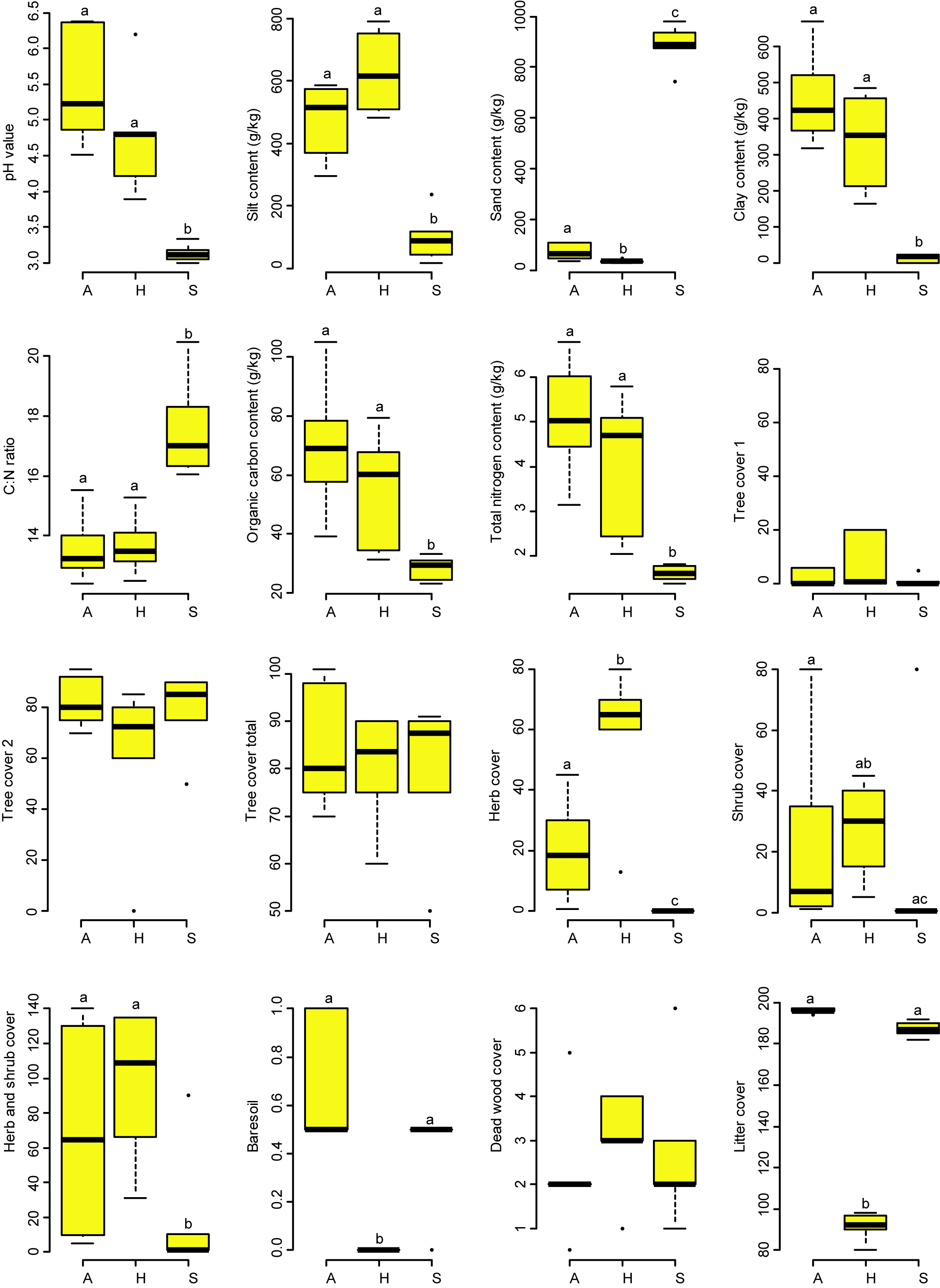

Supplement: Figure S1 — Soil and understory vegetation parameters among the three study regions depicted using box plots. Schwäbische Alb (A), Hainich-Dün (H) and Schorfheide Chorin (S) study sites. Different letters above bars indicate significant differences between the sites (p≤0.05) based on a Tukey post hoc pairwise comparison. (TIF) [file pone.0047500.s001.tif]

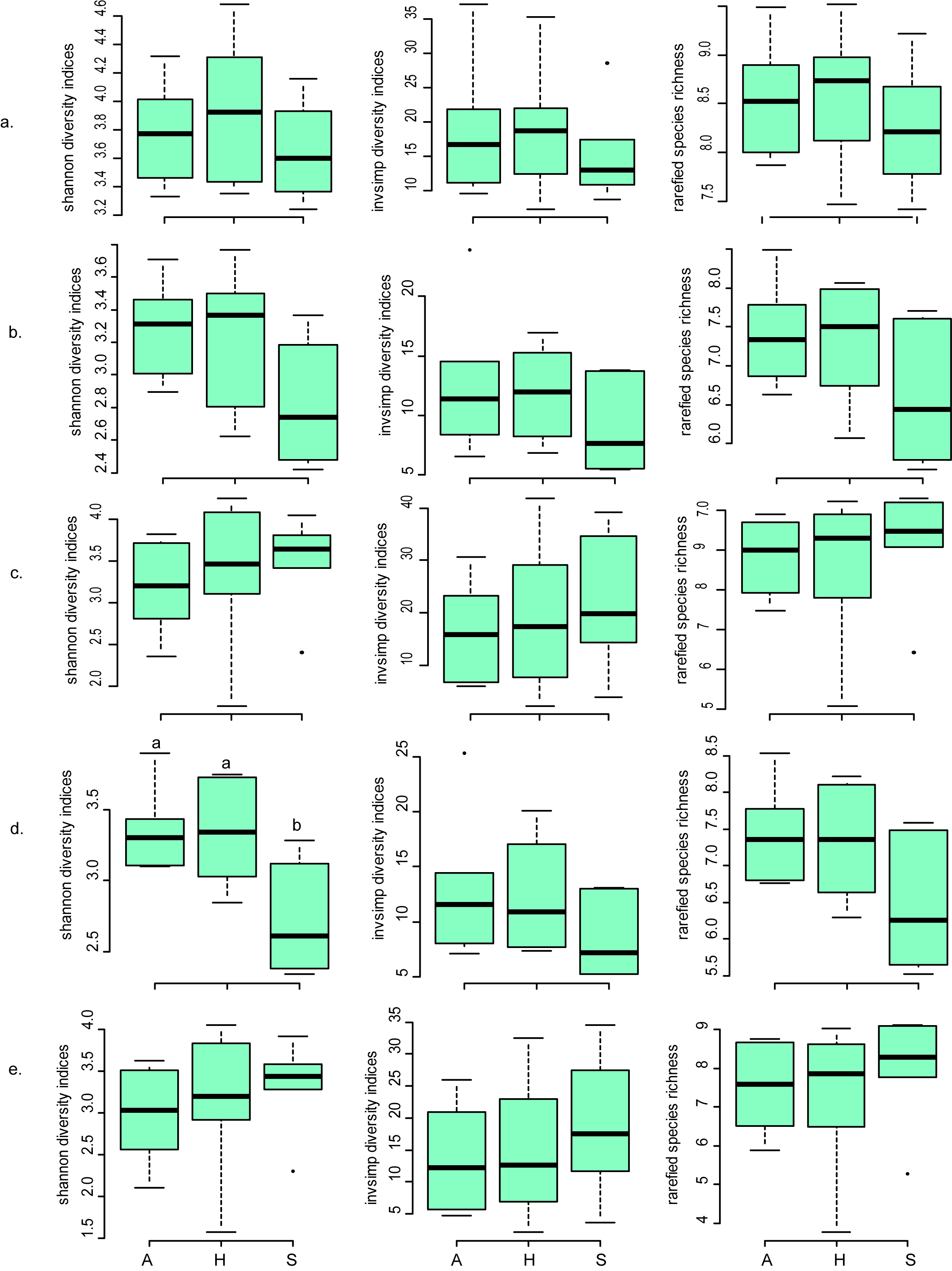

Supplement: Figure S2 — Rarefied species richness, Shannon and invsimp diversity indices across the three study regions, Schwäbische Alb (A), Hainich-Dün (H) and Schorfheide Chorin (S), for: (a) the fungal kingdom, (b) the phylum Basidiomycota, (c) the subphylum Agaricomycotina, (d) the phylum Ascomycota and (e) the subphylum Pezizomycotina. Based on a Tukey post hoc pairwise comparison at p≤0.05 no significant differences were found. (TIF) [file pone.0047500.s002.tif]

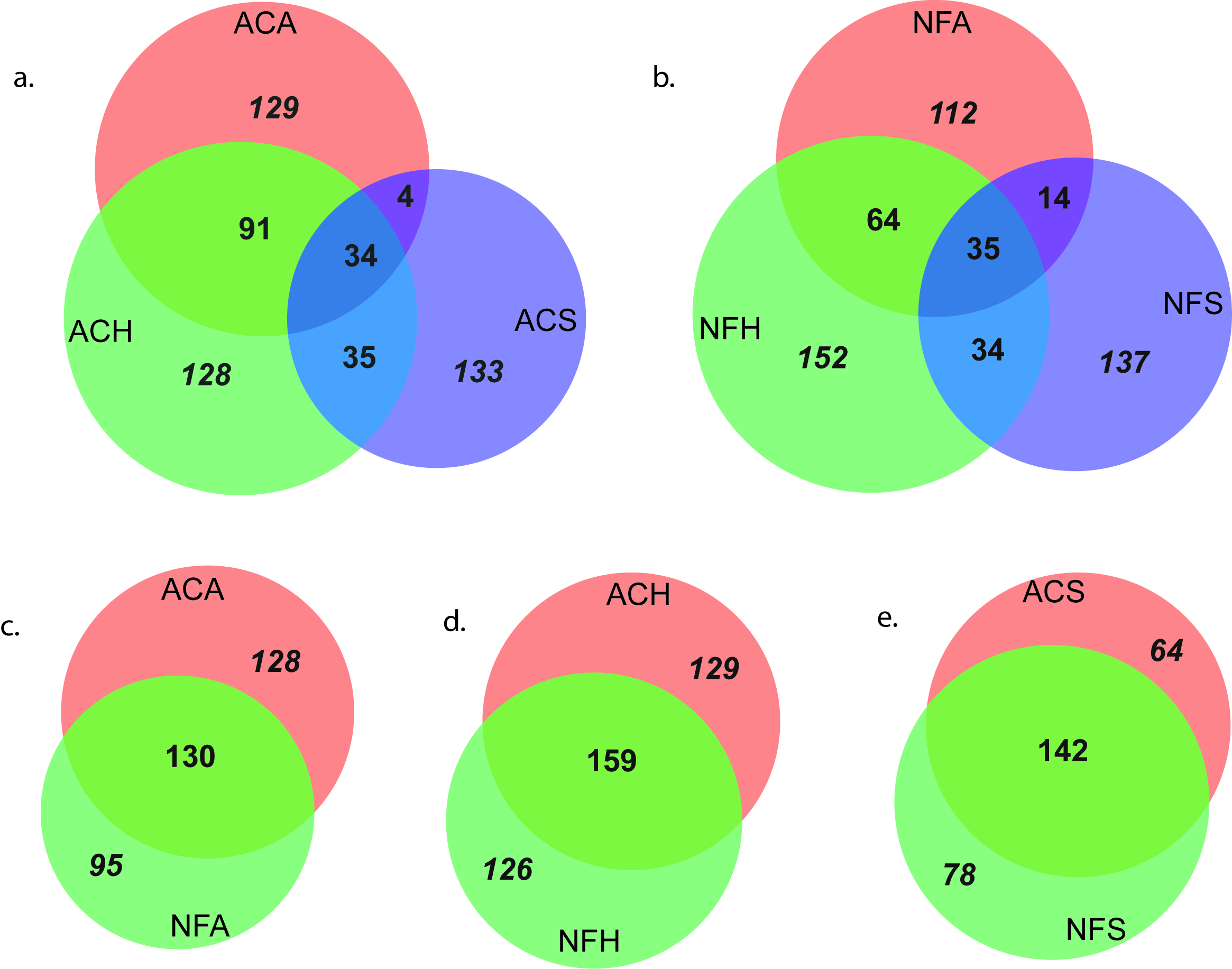

Supplement: Figure S3 — Distribution of shared and unique fungal OTUs among the two management types of the three study regions (a) age class beech forests (b) among the unmanaged beech forests, and between the two management types of the study regions Schwäbische Alb (c), Hainich-Dün (d) and Schorfheide Corin (e). (TIF) [file pone.0047500.s003.tif]

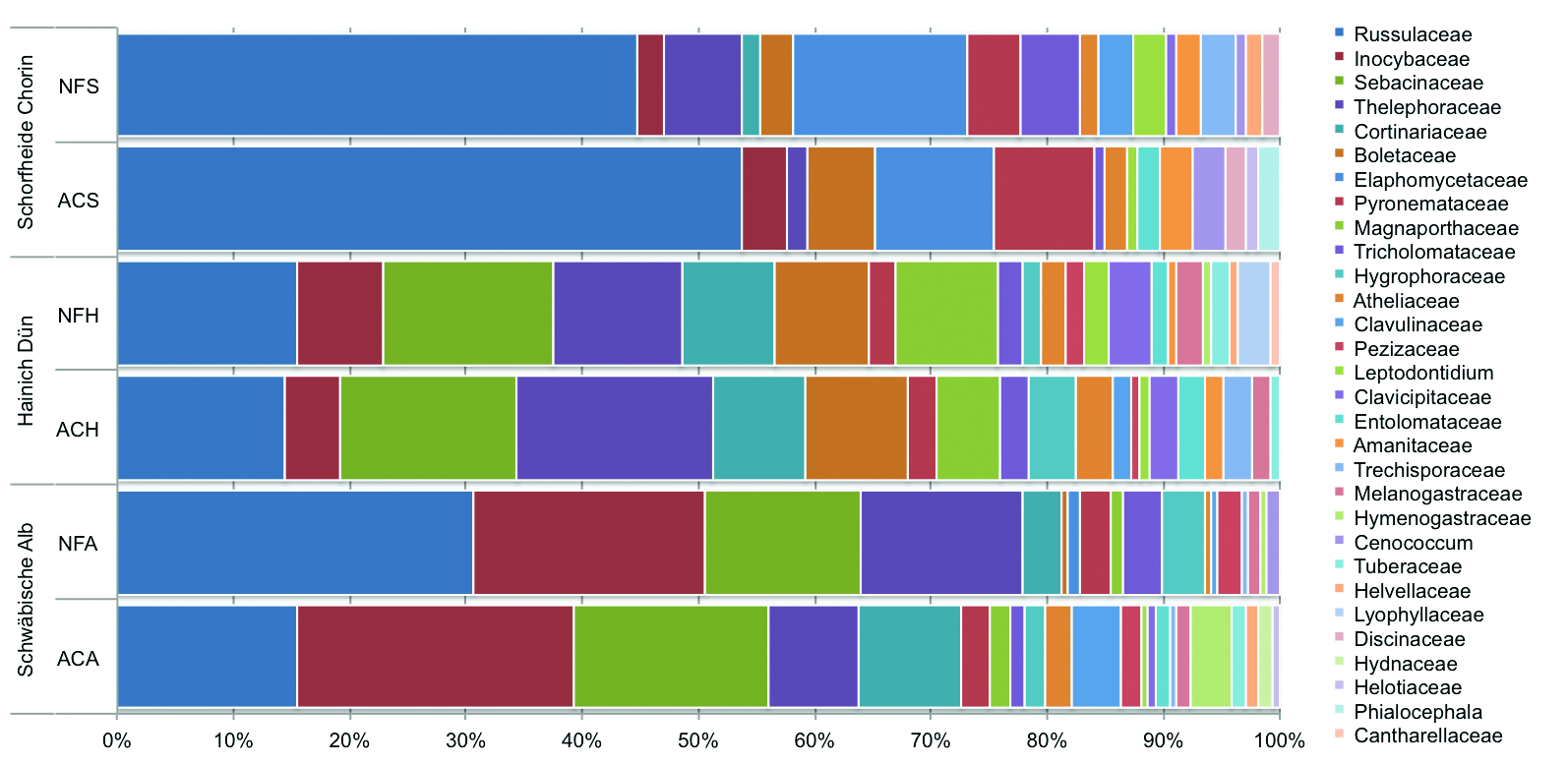

Supplement: Figure S4 — Relative distribution of ectomycorrhizal fungal families between the two management types in the three study regions. (TIF) [file pone.0047500.s004.tif]

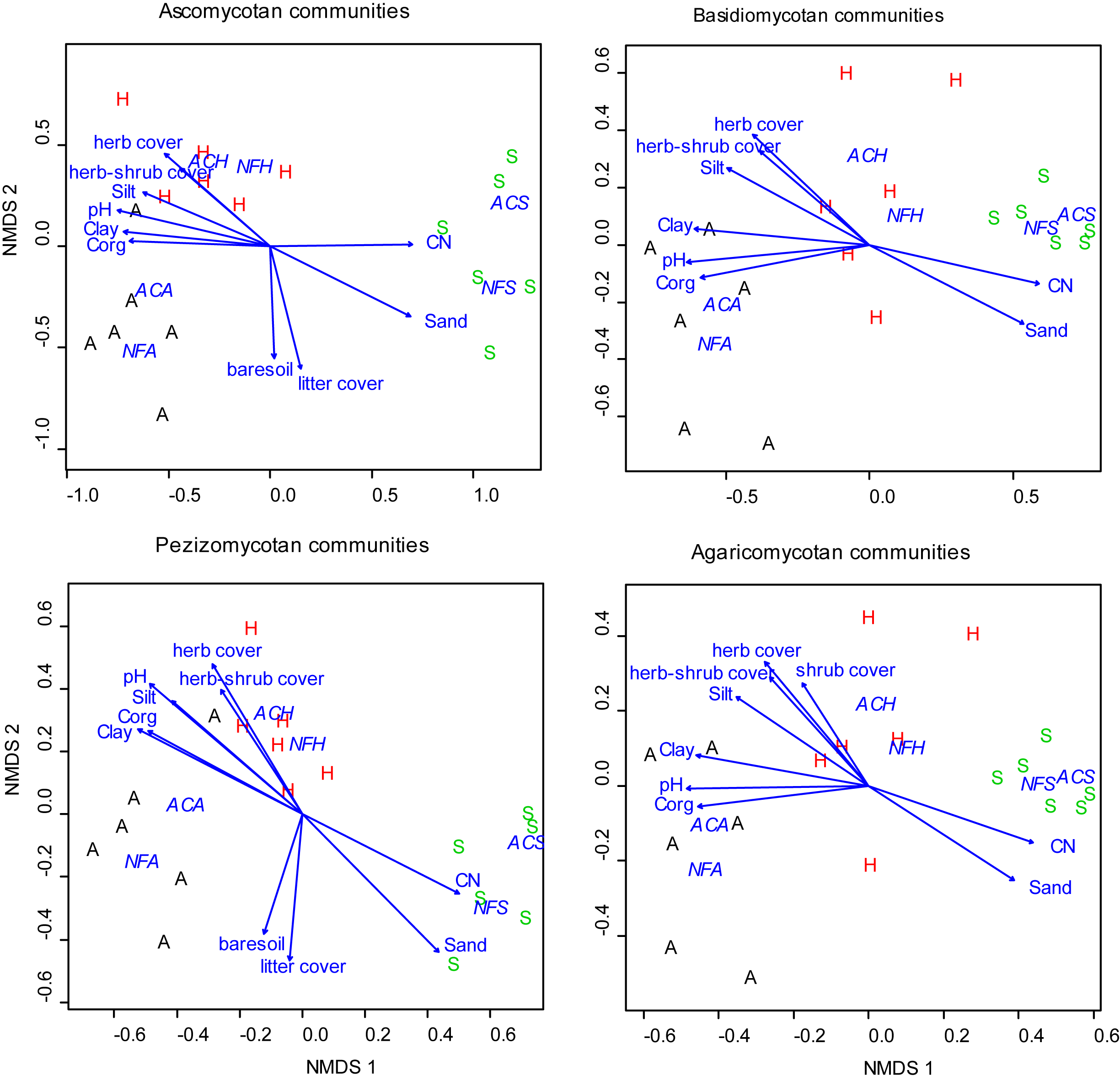

Supplement: Figure S5 — NMDs ordination of the study sites Schwäbische Alb (A), Hainich-Dün (H) and Schorfheide Chorin (S), based on the fungal community composition identified as members of the phylum Ascomycota (Ascomycotan communities, stress = 10.66), phylum Basidiomycota (Basidiomycotan communities, stress = 12.66), subphylum Pezizomycotina (Pezizomycotan communities, stress = 10.73) and subphylum Agaricomycotina (Agaricomycotan communities, stress = 12.80). Soil and plant parameters used as an explanatory variable and found to be significant (p≤0.05) are represented as vectors. The two management types are presented as AC = age class and NF = unmanaged beech forests followed by the respective study site abbreviations. (TIF) [file pone.0047500.s005.tif]
