# Supplementary material for: Differences in Soil Fungal Communities between European Beech (Fagus sylvatica L.) Dominated Forests Are Related to Soil and Understory Vegetation
Source: PLoS One. 2012 Oct 18;7(10):e47500. doi: 10.1371/journal.pone.0047500 (PMC3475711; doi:10.1371/journal.pone.0047500)
Supplement: Table S1 — Ectomycorrhizal fungal community distribution. Putative ECM fungal families and genera found in this study and the study sites and management types they are found. (Numbers refer to the number of fungal OTUs of the respective fungal family found in the respective management type). Abbreviations: AC = age class and NF = unmanaged beech forest. Note: For Ascomycetes fungal taxa without a clearly defined family classification we used the genus names in the family column. (DOCX) [file pone.0047500.s006.docx]

**Supporting information**

**Table S1** Ectomycorrhizal fungal community distribution. Putative ECM fungal families and genera found in this study and the study sites and management types they are found. (Numbers refer to the number of fungal OTUs of the respective fungal family found in the respective management type). Abbreviations: AC = age class and NF = unmanaged beech forest. Note: For Ascomycetes fungal taxa without a clearly defined family classification we used the genus names in the family column.

|  |  |  | | **Study sites and Management types** | | | | | | | |
| --- | --- | --- | --- | --- | --- | --- | --- | --- | --- | --- | --- |
|  |  |  | **Schwäbische Alb** | | | **Hainich-Dün** | | | **Schorfheide Chorin** | | |
| **Phylum** | **ECM family** | **ECM genera** | | **AC** | **NF** | | **AC** | **NF** | | **AC** | **NF** |
| Ascomycota | *Cenococcum* | Cenococcum | | 0 | 2 | | 0 | 0 | | 3 | 1 |
|  | Clavicipitaceae | Paecilomyces | | 1 | 0 | | 3 | 5 | | 0 | 1 |
|  | Discinaceae | Hydnotrya | | 0 | 0 | | 0 | 0 | | 2 | 2 |
|  | Elaphomycetaceae | Elaphomyces | | 0 | 2 | | 0 | 0 | | 11 | 20 |
|  | Helotiaceae | Hymenoscyphus | | 1 | 0 | | 0 | 0 | | 1 | 0 |
|  | Helvellaceae | Hervela | | 2 | 0 | | 0 | 1 | | 0 | 2 |
|  | Magnaporthaceae | Phialophora | | 3 | 2 | | 7 | 12 | | 0 | 0 |
|  | Pezizaceae | Hydnobolites, Pachyphloeus, Peziza, Sarcosphaera | | 3 | 4 | | 1 | 2 | | 0 | 0 |
|  | Pyronemataceae | Genea, Otidia, Pulvinula, Tarzetta, Wilcoxina | | 3 | 5 | | 3 | 2 | | 7 | 6 |
|  | Tuberaceae | Tuber | | 2 | 0 | | 1 | 2 | | 0 | 0 |
| Basidiomycota | Amanitaceae | Amanita | | 0 | 0 | | 2 | 1 | | 3 | 3 |
|  | Atheliaceae | Amphinema, Byssocorticium, Piloderma, Tylospora | | 4 | 1 | | 4 | 3 | | 2 | 2 |
|  | Boletaceae | Boletus, Xerocomus | | 0 | 1 | | 11 | 11 | | 6 | 4 |
|  | Cantharellaceae | Craterellus | | 0 | 0 | | 0 | 1 | | 0 | 0 |
|  | Clavulinaceae | Clavulina, Membranomyces | | 7 | 1 | | 2 | 0 | | 0 | 4 |
|  | Cortinariaceae | Cortinarius, Hebeloma | | 15 | 6 | | 10 | 11 | | 0 | 2 |
|  | Entolomataceae | Entoloma | | 2 | 0 | | 3 | 2 | | 2 | 0 |
|  | Hydnaceae | Hydnum | | 2 | 0 | | 0 | 0 | | 0 | 0 |
|  | Hygrophoraceae | Hygrophorus | | 3 | 7 | | 5 | 2 | | 0 | 0 |
|  | Hymenogastraceae | Hymenogaster | | 6 | 1 | | 0 | 1 | | 0 | 0 |
|  | Inocybaceae | Inocybe | | 40 | 37 | | 6 | 10 | | 4 | 3 |
|  | Melanogastraceae | Melanogaster | | 2 | 2 | | 2 | 3 | | 0 | 0 |
|  | Russulaceae | Gymnomyces, Lactarius, Russula | | 26 | 57 | | 18 | 21 | | 57 | 60 |
|  | Sebacinaceae | Sebacina | | 27 | 25 | | 15 | 20 | | 0 | 0 |
|  | Thelephoraceae | Pseudotomentella, Thelephora, Tomentella | | 13 | 26 | | 21 | 15 | | 2 | 9 |
|  | Tricholomataceae | Laccaria, Tricholoma | | 2 | 6 | | 3 | 3 | | 1 | 7 |
